# 13q14 Deletion and Its Effect on Prognosis of Chronic Lymphocytic Leukemia

**DOI:** 10.7759/cureus.16839

**Published:** 2021-08-02

**Authors:** Khizer Khalid, Jaskamal Padda, Mohammad Syam, Amir Moosa, Varsha Kakani, Sujana Sanka, Ujala Zubair, Sandeep Padda, Ayden Charlene Cooper, Gutteridge Jean-Charles

**Affiliations:** 1 Internal Medicine, JC Medical Center, Orlando, USA; 2 Internal Medicine, Avalon University School of Medicine, Willemstad, CUW; 3 Internal Medicine, Kakatiya Medical College, Warangal, IND; 4 Family Medicine, Dow University of Health Sciences, Karachi, PAK; 5 Internal Medicine, Advent Health and Orlando Health Hospital/JC Medical Center, Orlando, USA

**Keywords:** 13q14, b lymphocytes, microrna, chemotherapy, chronic lymphocytic leukemia (cll)

## Abstract

Chronic lymphocytic leukemia (CLL) is the most common leukemia affecting adults. CLL results due to uncontrolled accumulation of B lymphocytes in the body with the clinical spectrum ranging from comparatively benign disease to an aggressive form. The disease pathogenesis lies in molecular genetics, the most common alteration being the deletion in the long arm of chromosome 13, at position 14 (13q14) region. This deletion leads to the loss of important microRNAs which are involved in maintaining the critical balance of the apoptosis mechanism of cell death of B lymphocytes. As such, the imbalance contributes towards B cells' immortality and, thus, CLL arises. This significant 13q14 deletion contributes to CLL's pathogenesis and paves the way for CLL treatment, hence affecting the prognosis of the affected patients.

Furthermore, the size of deletion of the long arm of chromosome 13 (13q) has a remarkable effect on its prognosis and therapeutic intervention. The minimal deleted region (MDR)/small deletion or long 13q loss/mutation, and biallelic 13q deletion or monoallelic 13q deletion are commonly seen. 13q14 deletion is an initiating defect targeting tumor suppressor gene locus deleted in lymphocytic leukemia 2 (DLEU2))/microRNA15A (MIR15A)/microRNA 16-1 (MIR 16-1). Regarding CLL treatment, conventional therapy with alkylating agents has been used for a long time, which reported low- to non-existent complete remission rates and adverse events after prolonged use. Moreover, research into the 13q14 deletion has also provided new insights into the molecular genetics and pathways that interact in such a way, making it possible to transform healthy cells into malignant cells in an entirely new fashion with a complete disregard to its original form, resulting in CLL.

## Introduction and background

Chronic lymphocytic leukemia (CLL) is a tumor of B lymphocytes. It is characterized by the uncontrolled accumulation of B lymphocytes in the body, which usually results from apoptosis disruption [1]. CLL is a stage of small lymphocytic lymphoma. It is the most commonly seen leukemia in adults [2]. CLL exhibits a complete spectrum of clinical disease severity, ranging from a stable clinical disease with patients who can lead a near-normal life to patients with a malignant and proliferative disease who cannot expect to survive more than a few months. Besides this, different patients show different response rates to oncologic treatment. With time, CLL patients can transform into an acutely severe and aggressive form called Richter syndrome. This form presents similar to diffuse large B cell lymphoma, the most common type of non-Hodgkin’s in adults [3]. The severity of the disease and its clinical outcome is determined partly by the underlying molecular pathogenesis, and genome-wide analysis has identified multiple alterations in the coding region of the CLL genome which is responsible for the disease and its severity. However, the majority of CLL patients usually exhibit one of four chromosomal aberrations: 13q14 deletion, deletion of the long arm of chromosome 11, position 22-23 (11q22-23), deletion of the short arm of chromosome 17 at position 12 (17p12), and trisomy 12. Research has proven that 50-60% of CLL patients have the 13q14 deletion and it remains to be the most common genetic alteration which affects CLL, and this is supported by the fact that the deleted region codes for microRNAs miR15A and microRNA16A, which have an inhibitory effect on regulators of apoptosis [4,5]. With the loss of these microRNAs, that inhibitory effect is lost, leading to the immortality of the faulty B cells and, thus, paving the way for CLL development. B cell lymphoma 2 (BCL2) gene is the one, among others, that is upregulated in CLL patients who show 13q14 deletion in their genome [4]. BCL2 gene codes for an antiapoptotic protein and its prime role in CLL contribution have been used by the drug called venetoclax, which targets BCL2 leading to a good response in the CLL patients [6]. 

## Review

Molecular genetics 

Chromosomes are made of DNA and the gene is a product of DNA; 13 chromosomes contain numerous genes responsible for multiple disease development. Repeated mutations of genes are a distinctive feature of human malignancy. Gene mutation itself is a continuous process, and high-frequency mutation happens in few genes compared to the rest of the genes which undergo low-frequency mutation. Almost 80% of CLL exhibits chromosomal abnormalities. CLL patients show alterations in one out of four deletions of the long arm of chromosome 11 (11q), long arm of chromosome 13 (13q), short arm of chromosome 17 (17p) chromosomes, and trisomy 12, which have essential prognostic value with a contributory role in CLL pathophysiology and therapeutic intervention [7]. The deletion of 13q is the most common abnormality in CLL [8]. Solitary mutation of the 13q chromosome has the most favorable outcome. These genome alterations are identified by fluorescence in situ hybridization (FISH). The size of the 13q deletion affects its prognosis [9,10].

The MIR15A/MIR16-1 cluster acts as the tumor suppressor gene in CLL. It is an example of an MDR which is incorporated in the DLEU2 gene and microRNA [11]. Deletion limited to MIR15A/MIR 16-1 is better than DLEU2 associated region involvement in terms of prognostic value [8]. A more aggressive clinical course is reported in large segment 13q deletion attached with retinoblastoma 1 (RB1) gene, which is considered as tumor suppressor gene. As a result, it shows a transient treatment response and less survival time than type 1 deletion. Furthermore, a significant 13q loss downregulates ten other genes, including translationally controlled tumor protein, accountable for survival and growth signaling via suppression of BCL-2 associated X-protein (BAX) related apoptosis and tumor protein p53 genes [12]. Also, deregulation of many relevant cellular pathways is linked to higher percentages of 13q deletion [7]. The more deletion increases, the more progressive disease ensues. However, some studies dispute this opinion [7,8].

In contrast, biallelic losses in 13q are observed in 30% of cases. These trivial deletions do not involve the RB1 gene and are associated with more clinical aggressive trials. Nonetheless, some authors hypothesized that monoallelic 13q deletions occur in the initial stage, reveal large deletion, and are related to more aggressive outcomes. The outcome of allelic deletion has been controversial. Hence, it is assumed that the prognosis effect of biallelic mutation may be masked by the size of the deletions or inactivation of the rest allele by other processes [7]. Since the polymorphism of 13q deletion is apparent, recent studies suggest that isolated 13q deletion is considered a low-risk group that reacts remarkably to conventional alkylating agents such as fludarabine and chlorambucil, which have promising progression-free survival [7].

The incidence of genetic abnormalities in CLL

13q14 deletion is detected in more than 50% of CLL patients with excellent follow-up with <1% progression yearly [4]. 11q23 deletion is linked to the ataxia-telangiectasia mutated gene and is detected in 5-20% CLL patients. It is highly variable in size and develops in 30% of disease prolapse [4]. Trisomy 12 is observed in 10-20% of CLL cases and is associated with other chromosomal deformities [7]. 17p deletion at position 13 is noticed in approximately 3-8% of CLL patients and is responsible for frequent refractory CLL cases up to 30% [7]. In addition to that, splicing factor 3B subunit 1 and notch homolog 1 translocation-associated mutations are reported in 10-15% of newly diagnosed CLL cases [4]. The prognostic role of some low-frequency mutant genes like baculoviral IAP repeat-containing 3, sterile alpha motif and HD domain 1, ribosomal protein S15, nuclear factor of kappa light polypeptide gene enhancer in B-cell inhibitor epsilon, early growth response 2, K-ras protein-oncogene, and proton-dependent oligopeptide transporters is still debatable and demands more studies to establish accountability [8].

Diagnosis

The initial diagnostic evaluation of CLL includes a complete blood count with differential, peripheral blood smear, and immunophenotyping. For a CLL diagnosis to be made, there should be ≥5000 B-cells/μl present in the blood for three months. Flow cytometry helps in determining the clonality of the disease [13]. FISH is very useful to detect the type of abnormality associated with the particular case of CLL like 13q deletion, trisomy 12, 11q deletion, 17p deletion, etc. [14]. FISH not only detects the 13q14 deletions but also calculates the deletion load [8]. Rai and Benet's staging was used for a long time to know the prognosis and aided in managing the disease. These conventional staging techniques lagged in identifying patients in the initial stages of the disease who will progress to need treatment. However, the newer biological techniques now replace them and help in establishing a better prognosis and treatment. The new biological techniques include prognostic serum markers like soluble cluster of differentiation (CD) 23, cell surface proteins like CD38, cytoplasmic proteins like zeta chain associated protein kinase 70, and various cytogenetical studies [15,16]. 

Prognosis

As detected by FISH, the most common chromosomal abnormality associated with CLL is the deletion of 13q14, constituting approximately 50% of all cases. It generally has a good prognosis but some forms have led to unfavorable outcomes. This variation in the level of prognosis depends on the anatomic aspects of the deletions. The anatomical aspects constitute the size of the deletions, either small or large, monoallelic or biallelic, and deletion of 13q either in the telomeric region or the centromeric region. This difference plays a vital role in determining the clinical outcomes in each patient [17]. Isolated 13q deletion carries a better prognosis than combined mutations involving other chromosomes. The size of deletions in the long arm of chromosome 13 varies widely. Large 13q14 deletions are associated with aggressive tumors and poor outcomes compared to short deletions [8]. Large deletions or a high number of losses in 13q14 at the time of diagnosis are associated with low survival rates due to increased proliferation and reduced apoptosis. So, it is essential to determine the number of cells showing genetic abnormality. There are usually two types of deletions: type one constitutes the miR15A/16-1 deletion and type two constitutes the RB1 gene deletion [8]. In type one, the deletion of both these microRNAs has been shown to increase the multiplication of B lymphocytes by modifying the gene expression, which controls the cell cycle [18]. Type two is the deletion of the RB1 gene, a tumor suppressor gene located on chromosome 13. As it is a tumor suppressor gene, its loss leads to the development of different types of cancers like breast, lung, bone, and bladder [6]. Type one has a better prognosis compared to type two [8, 18]. There is a difference in the conclusion of various studies conducted to determine the prognostic outcomes of biallelic versus monoallelic deletions [8]. Biallelic loss accounts for 30% of all 13q deletions in CLL patients [19]. Orlandi et al. found that biallelic deletions constitute a bad prognosis as they had a significant deletion burden. However, their study mentioned that deletion type could not be considered an independent factor in determining the prognosis [20]. Another study from 2008 also mentioned that biallelic 13q deletions are aggressive, while few other studies showed no difference between biallelic and monoallelic groups [8]. According to a study, the median survival in CLL patients with 13q deletions was 133 months [19]. Hernandez et al. stated that the percentage of losses of 13q14 and B symptoms are the important factors determining the prognosis. 13q deletions involving less than 80% losses led to more prolonged survival of 163 to 254 months with a death proportion of 3.5%. More than 80% of losses are associated with low survival of 56 months and a death proportion of 22.2%. In addition to the independent factors like the percentage of losses and the presence of B symptoms, several other factors determine the overall survival. These include high serum lactate dehydrogenase levels, beta-2 microglobulin, level of infiltration of bone marrow, and splenomegaly [21].

Treatment

Since the past decade, the treatment of CLL has become more effective and challenging. Research into the microenvironment of CLL signaling pathways has been studied to develop new novel therapies. Many drugs have been approved by the US Food and Drug Administration (FDA) that can be used in the frontline or in a relapsed setting. These drugs may be used as single agents or in combinations. Frontline drugs include alkylating agents, purine analogs, anti-CD20 monoclonal antibodies, and Bruton tyrosine kinase (BTK) inhibitor. While drugs for relapse include ibrutinib, BCL-2 inhibitor (venetoclax) +/- rituximab, anti-CD20 monoclonal antibodies, and cellular therapies [22,23].

Conventional therapies used in CLL

Initially, chlorambucil was used as frontline and sole therapy for CLL. It was considered the "gold standard" and used for several decades. It has many advantages, such as oral route of administration, low cost, and low toxicity [14]. Fludarabine, pentostatin, and cladribine are the other purine analogs used in CLL. Combination therapy of cyclophosphamide plus fludarabine yielded promising results. Conventional modes of treatment are cyclophosphamide, doxorubicin, vincristine, prednisone (CHOP); cyclophosphamide, doxorubicin, prednisone (CAP); chlorambucil. Bendamustine was also used for CLL as monotherapy [13].

Novel therapies used in CLL

**Figure 1:**
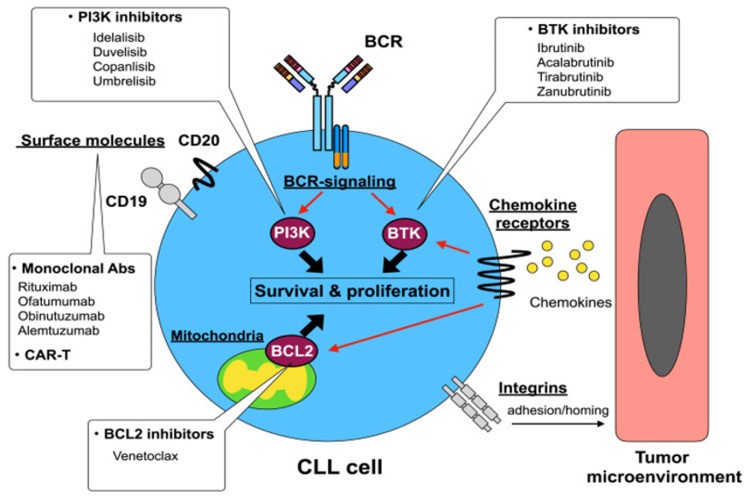
Novel drugs and their target molecules and pathways. PI3K, phosphoinositide 3 kinases; BCR, B cell receptors; BTK, Bruton tyrosine kinase; BCL2, B cell lymphoma 2; Abs, antibodies; CAR-T, chimeric antigen receptor T cell; CLL, chronic lymphocytic leukemia Copyright/License Licensee The JSLTR, Japan. This figure is from an open-access article distributed under the terms and conditions of the Creative Commons Attribution-NonCommercial-ShareAlike 4.0 International License. (https://creativecommons.org/licenses/by-nc-sa/4.0/deed.en) No modifications were made to the original figure [24].

Development of novel therapies targeting various signaling pathways was possible after studying the pathogenesis of CLL with regard to recurrent mutations, and its immunological aspects. Figure 1 states the various novel therapies and their site of action [24].

Anti-CD20 Antibodies

Rituximab is an anti-CD20 chimeric monoclonal antibody used in high doses as monotherapy [14]. The combination of rituximab, cyclophosphamide, and fludarabine is highly effective compared to rituximab monotherapy against CLL [24]. Another human monoclonal antibody that targets the proximal epitope of CD20 is ofatumumab, which is effective against fludarabine refractory CLL patients [13,24]. Obinutuzumab is another humanized, fucosylated type II antibody that is used in heavily pretreated relapsed/refractory CLL [24]. Alemtuzumab acts against CD52 antigen and is humanized monoclonal antibody. It is indicated in patients with advanced CLL who failed/relapsed with fludarabine therapy and also effective against the CLL patients with deletions of chromosome 11 or 17 [13].

In a CALGB 9011 trial, patients receiving a combination of rituximab and fludarabine reported better results than using fludarabine alone [13]. In the CLL8 trial, the combination of cyclophosphamide and rituximab reported good results in CLL patients, particularly with mutated immunoglobulin variable heavy chain, deletion of 13q, trisomy 12, or deletion of 11q. They also achieved durable remission and a good survival rate following treatment with fludarabine, cyclophosphamide, and rituximab [13].

Inhibitors of B-Cell Receptor and Chemokine-Receptor Signaling

These drugs act at the intracellular kinases and interfere with the B cell stimulation. So, the capacity of lymphocytes to recirculate between the blood environment and the protective microenvironmental niches in lymphoid tissue decreases. Hence immediately, we can see rapid shrinkage of lymph nodes and lymphocytosis [22].

Ibrutinib and Second Generation Brutinibs That Inhibits BTK

Ibrutinib is a BTK inhibitor and acts by forming a covalent bond to the cysteine-481 residue of BTK [24]. It is recommended as initial single-agent therapy to the CLL patients by National Comprehensive Cancer Network Committee. It should be continued until resistance develops or intolerance to the therapy [22]. A combination of ibrutinib and rituximab is used in high-risk CLL patients [14]. The second generation BTKs are acalabrutinib, tirabrutinib, zanubrutinib, vecabrutinib. These drugs don't require a covalent bond and are used in cysteine to serine substitution mutation in BTK [22].

Idelalisib and Second-Generation Lisibs That Inhibit Phosphoinositide 3 Kinases

These drugs inhibit the delta isoform of PI3K which causes early reduction of lymph nodes and concomitant lymphocytosis [22]. Idelalisib is initially approved by USFDA to be used in combination with the rituximab. But due to hepatotoxicity, increased incidence of infections, and deaths in CLL patients, the drug is no longer recommended [22]. Duvelisib is a dual PI3K gamma, and PI3K delta inhibitor drug and is used in patients with relapsed/refractory CLL [24]. Another PI3K inhibitor, the umbralisib drug, has clinical activity in combination with ibrutinib [22].

BCL-2 Inhibitor

BCL-2, an antiapoptotic protein, is seen in increased amounts in CLL patients. This protein acts against pro-apoptotic proteins such as BAX with their specific BCl-2 homology 3 (BH3) domains. Venetoclax is a BH3 mimetic drug that inhibits BCL-2 of leukemic cells and induces apoptosis. This drug is highly efficacious in relapsed /refractory CLL patients, particularly with the deletion of 17p mutations [13,23]. Promising results were reported when the combination of venetoclax and rituximab is used [13]. A combination of venetoclax and obinutuzumab was approved as initial therapy by FDA and National Comprehensive Cancer Network Committee guidelines [22].

Pembrolizumab and Checkpoint Inhibition

The programmed death-one pathway is essential for blocking the immune surveillance of CLL and is highly expressed in pretreated tumor cells. Pembrolizumab is a programmed antibody that was used in patients with relapsed and Richter transformation type of CLL. It was found that there is a benefit in the blockade of the program death-one pathway in these patients [13].

Chimeric Antigen Receptor T Cells 

T cells have a chimeric antigen receptor specifically to CD19. CD19 is a B cell antigen coupling along with CD137 that is a co-stimulatory receptor in T-cells and CD3 zeta which is a signal transduction component of the T-cell antigen receptor [14]. The therapy has been developed to target CD19 and is initially used in CLL. However, due to the side effects of cytokine release syndrome and neurotoxicity, chimeric antigen receptor (CAR) natural killer cells therapy is employed now. CAR natural killer cells therapy demonstrated rapid and persistent efficacy against CLL and did not report any significant side effects [13,24].

Drugs Targeting Receptor Tyrosine Kinase-Like Orphan Receptor 1

Receptor tyrosine kinase-like orphan receptor 1 is an onco-embryonic surface antigen that acts as a receptor for the Wnt family member 5a signaling pathway and is found in high levels in CLL patients compared to normal healthy adults. Cirmtuzumab is a monoclonal antibody that targets this pathway with high affinity and high specificity. It was evident from the preclinical studies that a combination of ibrutinib and cirmtuzumab is more effective than treatment with either agent alone [22].

Lenalidomide

Lenalidomide is a thalidomide analog and demonstrated favorable results in the treatment of high-risk CLL patients, including 17p deletion carriers. CLL patients on this drug reported a "tumor flare reaction," which causes heat and burning sensation in lymph nodes [13]. Combination therapy of lenalidomide, rituximab, and fludarabine was initially considered in untreated CLL patients, but the trial has closed prematurely due to the high toxicity and low response rate [13].

## Conclusions

The most common leukemia in adults is CLL which is a neoplasm of B cell lineage. The deletion of 13q is the most common chromosomal abnormality accounting for nearly 50% of the cases. Isolated 13q deletion is associated with a better prognosis and clinical outcomes. However, there is a wide range of variation in the clinical course and outcome even within 13q deletions, which is attributed to the anatomical variations associated with the deletion. Large 13q14 deletions are associated with low survival rates compared to the more minor deletions. FISH is used to determine the cytogenetics of the disease. Determining the cytogenetics and deletion burden is of prognostic value and aids in determining treatment options. Conventional therapies with alkylating agents have been used for an extended period of time. Despite the advantages, such as low cost and low toxicity, they reported low to non-existent complete remission rates and severe side effect profiles. Research into the recurrent mutations, understanding the CLL microenvironment, and learning about the various signaling pathways has led to many novel therapies. Despite the development of many drugs and therapies, challenges continue, regarding the side effects and resistance to the therapies. Further larger medical trials are recommended to ensure the use of drugs that are safer and help in maintaining remission.
